# A preliminary study showing no association between methylation levels of C3 gene promoter and the risk of CAD

**DOI:** 10.1186/s12944-018-0949-4

**Published:** 2019-01-05

**Authors:** Gaojun Cai, Zhiying Huang, Lei Yu, Li Li

**Affiliations:** 1 Department of Cardiology, Wujin Hospital affiliated with Jiangsu University, Changzhou, Jiangsu Province, 213017 China; 2 Department of Pediatrics, No. 2 Hospital of Changzhou, Changzhou, 213001 Jiangsu China

**Keywords:** Complement component 3, Coronary artery disease, Methylation, Epigenetics

## Abstract

**Objective:**

Coronary artery disease (CAD) is a multi-factor disease. Complement component 3 (C3) plays an important role in the development of CAD. The present study investigated the association between DNA methylation status of *C3* gene promoter and the risk of CAD.

**Methods:**

One hundred CAD patients and 1 hundred age-and gender- matched controls were recruited in current study. Methylation levels in CpG island in *C3* promoter were determined by the method of bisulfite amplicon sequencing.

**Results:**

Methylation levels of four CpG sites in *C3* promoter were measured. There were no significant difference in methylation level of each CpG site between CAD patients and controls. Average methylation rate was also calculated. No significant difference in average methylation rate was observed between CAD and control groups. Stratified analyses based on EH, DM and smoking status were carried out, no significant association between *C3* promoter methylation levels and the susceptibility of CAD was observed. Furthermore, seven haplotypes were established and no significant difference in haplotypes was observed between CAD and control groups. However, our study showed that *C3* DNA methylation levels were positively associated with LDL-C levels.

**Conclusion:**

The present study showed no association between methylation levels of *C3* promoter and the risk of CAD. However, the methylation levels might be related to LDL-C levels.

**Electronic supplementary material:**

The online version of this article (10.1186/s12944-018-0949-4) contains supplementary material, which is available to authorized users.

## Introduction

Coronary artery disease (CAD) remains the leading cause of morbidity and mortality worldwide. With the rapid aging of the population, increased calorie consumption and decreased physical activity, the incidence of cardiovascular disease in some developing countries will increase steadily in future [1]. The interaction among environmental, genetic and epigenetic factors affects the occurrence and development of CAD, which has been accepted widely [2].

Epigenetic regulation, changing the expression of genes without changing the DNA sequence, plays an important role in the occurrence of various diseases. In recent years, the research on the association between epigenetics and cardiovascular disease was investigated more and more deeperly [3]. Candidate genes and epigenome-wide association studies (EWAS) showed that DNA methylation, the most common and important epigenetic modification pattern, was related to cardiovascular disease [4–6].

Complement component 3 (C3), one of the important components of complement system, is the central molecule of complement activity pathway [7]. C3 is mainly synthesized and secreted by liver and adipose tissues, composing of α and β chains. The *C3* gene is located in human chromosome 19p13.3–2. Several polymorphism sites in *C3* have been associated with CAD. Previous studies have showed that the activity of C3 was associated with several CAD risk factors [7], such as essential hypertension, diabetic mellitus and obesity. In 2014, Jiang H et al. found that C3 level was associated with the occurrence and development of CAD [8]. Inhibiting the interaction between C3 and fibrinogen could reduce the cardiovascular events in diabetic patients [9]. The association of *C3* methylation and body mass index (BMI) has been previously reported in Spanish population [10]. However, up to now, no study has designed to explore the relationship between *C3* promoter methylation and the CAD risk. Herein, we performed this case-control study to investigate whether *C3* methylation was associated with the risk of CAD and lipid profiles in a Chinese Han population.

## Materials and methods

### Study population

One hundred CAD patients (66 males and 34 females, mean age 64.1 ± 10.9 years) and 1 hundred age-and gender- matched controls (54 males and 46 females, mean age 63.1 ± 8.2 years) were recruited from the Department of Cardiology in Wujin Hospital affiliated to Jiangsu University between September 2013 to June 2017. The method of propensity score matching was used to select the subject. All subjects underwent coronary angiography (CAG) examination. The diagnosis of CAD was described in our previous studies [11]. CAD severity was substituted by the number of stenotic coronary artery. CAD patients were divided into three groups according to the number of stenotic coronary artery (one, two and three). The controls were who had angina-like chest pain and also underwent CAG examination in the same period. According to the results of CAG, controls had a luminal stenosis of *<* 50% in the major coronary arteries. As a result of the destruction of some serum samples, among the 200 participants, only 44 subjects were selected to detect for serum C3 levels, including 31 CAD patients and 13 controls. People with asthma, malignancy, serious kidney or hepatic disease were excluded. Patients taking lipid-lowering drugs in 3 months prior to the study, which might affect the lipid metabolism, were also excluded from the study.

The study protocol was approved by the Ethics Committee of our hospital and informed consent was obtained from all enrolled patients.

### Biochemical analysis

After overnight fasting, venous blood was drawn to detect biochemical data. The methods of detection of lipid levels, including TC, triglyceride (TG), high density lipoprotein cholesterol (HDL-C) and LDL-C, apolipoprotein A1 (Apo A1) and Apo B, were described in our previous studies [11]. Serum C3 levels were detected by radio-immunoassay method.

### DNA extraction and bisulfite amplicon sequencing

Genomic DNA was extracted from whole blood. The methods were performed as previously described [12]. The quality and concentration of genomic DNA were assessed by gel electrophoresis and Nanodrop 2000 spectrophotometer (NanoDrop technologies, Wlimington, DE, USA). The concentration of DNA was more than 20 ng/μL and the 260/280 absorbance ratio was between 1.8~2.0.

CpG islands in the promoter of *C3* gene were selected according to the following criteria: 1) 200 bp minimum length; 2) > 50%CG contents; 3) > 0.60 observed/expected the ratio of CpG dinucleotides. Only one region with four CpG sites met the inclusion criteria and was involved in the study. The detailed information of the four CpG sites lists in Additional file 1: Table S1. CpG sites were named as their relative distance to transcriptional start site (TSS).

The method of bisulfite amplicon sequencing was carried out to measure DNA methylation levels. Bisulfite conversion was performed using the EZ DNA Methylation-Glod Kit (ZYMO, CA, USA) according to manufacturer’s standard protocol, which was reported by Zhou SY et al. [13]. The bisulfite conversion rate of each sample was ≥99% in our study and no significant difference was found between CAD and controls groups. The primers used for polymerase chain reaction (PCR) were designed by Primer 3 software (http://primer3.ut.ee/) and the information of primers was shown in Additional file 2: Table S2. After PCR amplification (HotStar Taq polymerase, TaKaRa, Dalian, China) and library construction, products were sequenced on Illumina Hiseq 2500 Benchtop Sequencer (CA, USA). All samples achieved a mean coverage of >1000X. Q30 of each sample was more than 98.22%.The calculating methods of methylation level of each CpG site and average methylation level of CpG region were reported by previous study [14].

### Statistical analysis

Normality of quantitative variables was assessed by Kolmogorov-Smirnov test. If the data was normal distribution, it was presented as mean ± standard deviation (SD) and compared using an independent samples *t* test; otherwise, it was presented as median (interquartile range) and compared using Mann-Whitney *U* test. Multivariable logistic regression was used to determine associations between CpG sites in *C3* and CAD, which was adjusted for smoking, EH and DM. Subgroup analyses based on status of smoking, EH and DM were carried out. And the results were adjusted by Bonferroni correction (for average methylation, corrected *P <* 0.017; for each CpG site, corrected *P <* 0.004). Categorical variables were expressed as frequencies and percentages and compared using a Chi-square test. Person correlation analysis was used to evaluate the correlation between methylation level and lipid profiles. The haplotypes of CpG sites in *C3* gene were counted directly, according to the results of detection at the CpG site on the sequencing fragment. All data were analyzed using SPSS 17.0 software package (SPSS Inc., Chicago, Illinois). A two-sided *p*-value < 0.05 was considered statistically significant.

## Results

### Baseline characteristics

Table 1 shows the baseline characteristics of involved CAD patients and controls. Compared to controls, LDL-C levels in CAD patients were significantly higher (2.71 ± 0.75 vs 3.04 ± 0.98 mmol/l, *P* = 0.009); whereas, HDL-C and ApoAI levels in patients were significantly lower than those in controls.

**Table 1:** Baseline characteristics of involved CAD patients and controls

Characteristics	CAD (*n* = 100)	Control (*n* = 100)	*P*
Age, years	64.1 ± 10.9	63.1 ± 8.2	0.452
Male, n (%)	66 (66)	54 (54)	0.112
EH, n (%)	66 (66)	61 (61)	0.557
DM, n (%)	26 (26)	16 (16)	0.118
Smoking, n (%)	44 (44)	32 (32)	0.109
TC, mmol/l	4.70 ± 1.16	4.44 ± 0.81	0.064
TG, mmol/l	1.57 (1.06–2.40)	1.37 (0.96–2.11)	0.118
HDL-C, mmol/l	1.06 ± 0.23	1.20 ± 0.33	< 0.001
LDL-C, mmol/l	3.04 ± 0.98	2.71 ± 0.75	0.009
ApoAI, g/l	1.17 ± 0.20	1.31 ± 0.25	< 0.001
ApoB, g/l	1.00 ± 0.32	0.94 ± 0.22	0.081

*CAD* coronary artery disease, *EH* essential hypertension, *DM* diabetes mellitus, *TC* total cholesterol, *TG* triglyceride, *HDL-C* high-density lipoprotein cholesterol, *LDL-C* low-density lipoprotein cholesterol

### CpG methylation levels of *C3*promoter in CAD and controls

**Fig. 1:**
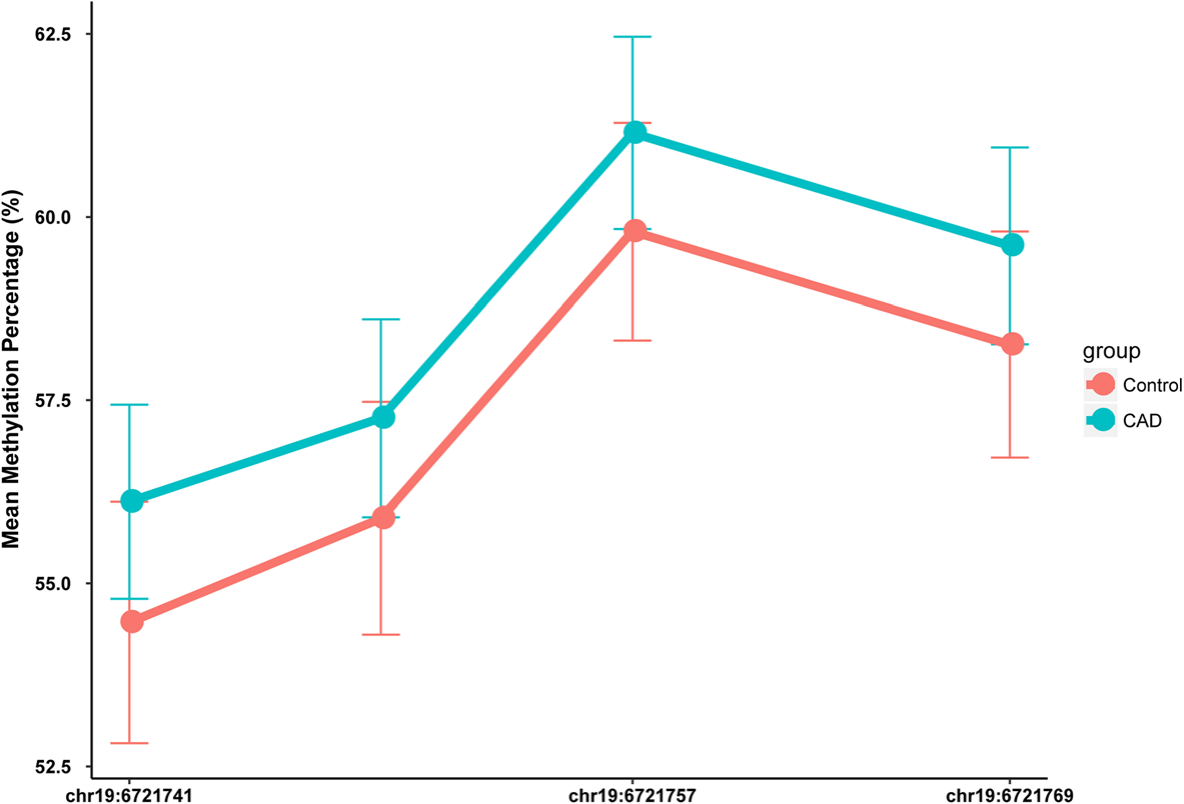
The methylation status of four CpG sites of *C3* promoter. The x-axis represents the genomic positions of the CpG sites; The y-axis represents the mean methylation percentage; The red line represents the control group; The blue line represents the CAD group

**Fig. 2:**
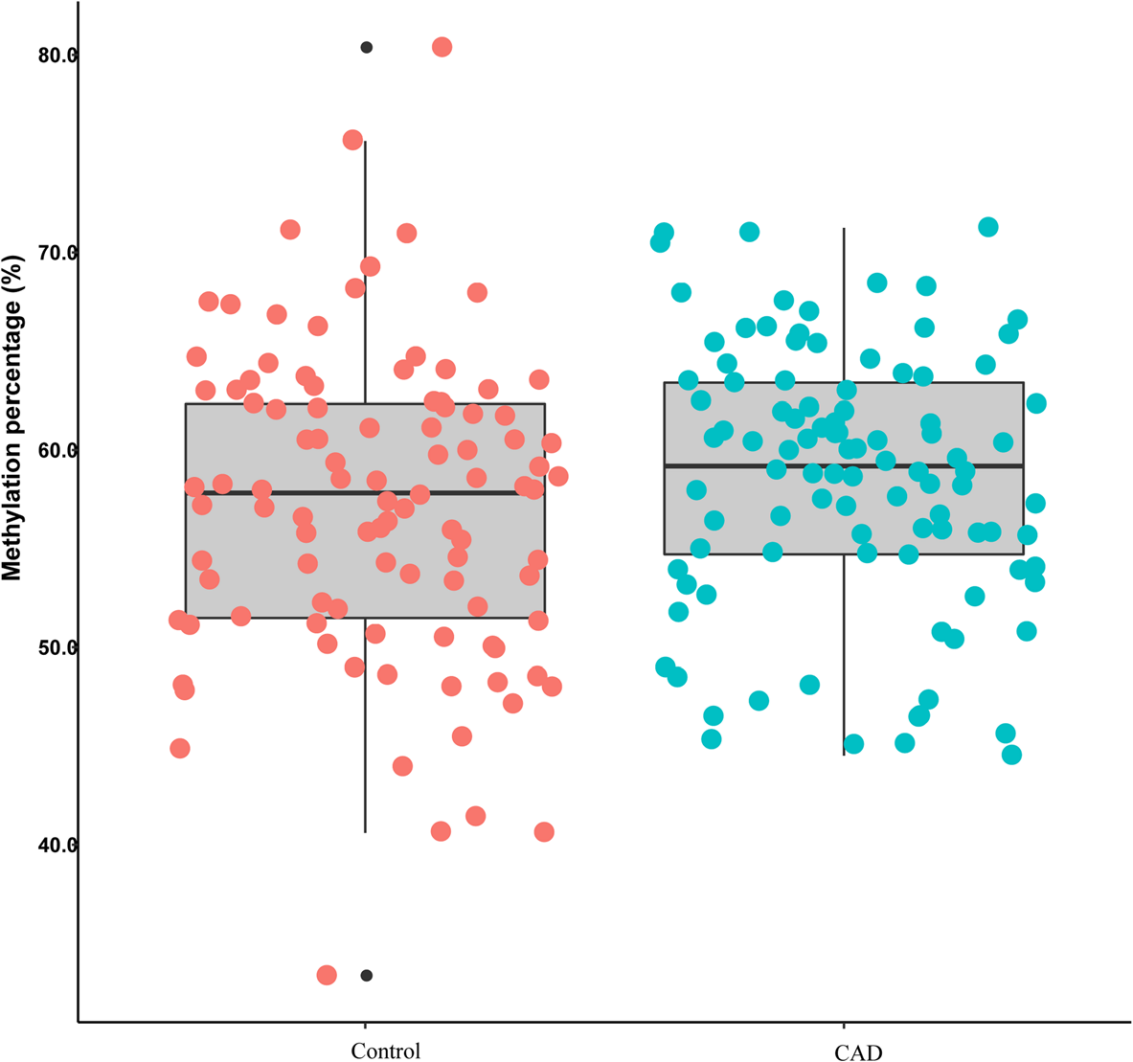
The mean methylation status of *C3* promoter

Firstly, methylation levels of four CpG sites located at the *C3* promoter locus were assessed (Table 2, Fig. 1). There were no significant difference in methylation level of each CpG site between CAD patients and controls. Average methylation rate was also calculated. No significant difference in average methylation rate was observed between CAD and control groups (Fig. 2). CAD severity was substituted by the number of stenotic coronary artery. CAD patients were divided into three groups according to the number of stenotic coronary artery (one, two and three). There was no difference between CpG sites methylation and CAD severity (data not shown). Stratified analyses based on EH, DM and smoking status were carried out, no significant association between *C3* promoter methylation levels and the susceptibility of CAD was observed (data showing in Additional file 3: Table S3-S5).

**Table 2:** Methylation levels of four CpG sites in *C3* promoter between CAD and controls

CpG site	CAD (%)	Control (%)	*P*	*P* ^a^
1	56.1 ± 6.7	54.5 ± 8.3	0.124	0.123
2	57.3 ± 6.8	55.9 ± 8.0	0.195	0.174
3	61.1 ± 6.6	59.8 ± 7.5	0.180	0.175
4	59.6 ± 6.8	58.6 ± 7.8	0.192	0.169
Average	58.5 ± 6.6	57.1 ± 7.8	0.165	0.153

*C*3 component 3, *CAD* coronary artery disease, *P*^a^ adjustment for EH, DM, and smoking status

### Haplotype of CpG sites between CAD patients and controls

Total seven haplotypes were detected (Table 3). The haplotypes were not significantly different between CAD and control groups.

**Table 3:** Haplotype of CpG sites between CAD patients and controls (%)

Haplotype	Depth	CAD (%)	Control (%)	*P*
CCCC	79,034	49.2 ± 5.9	47.8 ± 7.2	0.135
TTTT	60,288	34.2 ± 6.6	35.4 ± 7.6	0.179
TCCC	5947	3.6 ± 1.2	3.8 ± 2.3	0.671
TTCT	4449	2.5 ± 0.8	2.6 ± 0.8	0.358
CTCC	4221	2.7 ± 0.9	2.6 ± 0.9	0.283
CCCT	3688	2.3 ± 1.0	2.2 ± 0.7	0.890
CCTC	3666	2.3 ± 0.7	2.3 ± 0.7	0.772

*CAD* coronary artery disease

### Methylation levels of *C3* promoter and serum lipid profiles

**Fig. 3:**
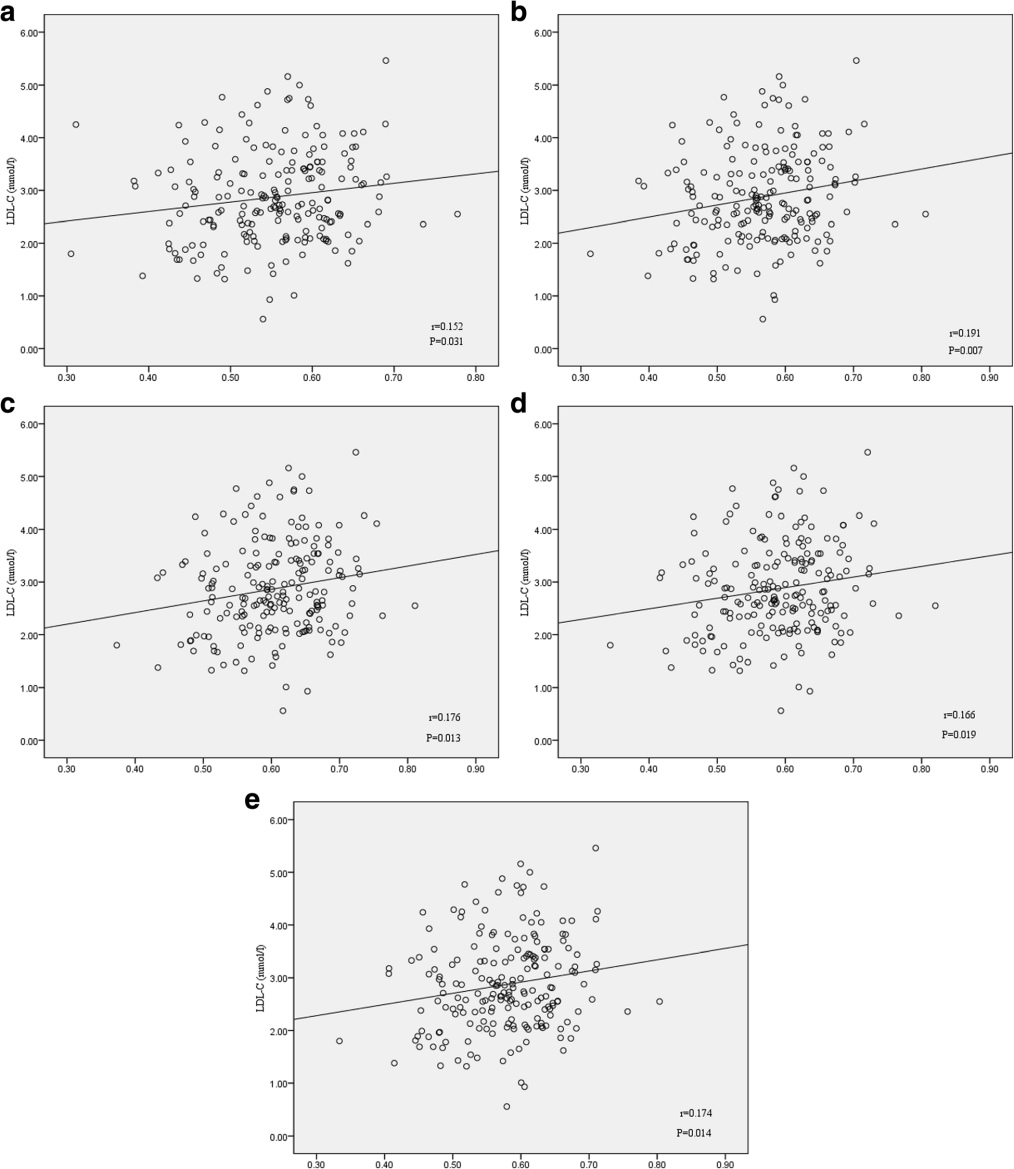
Pearson’s correlation between *C3* DNA methylation levels and LDL-C levels. **a** CpG site 1; **b** CpG site 2; **c** CpG site 3; **d** CpG site 4; **e** Average CpG sites

**Fig. 4:**
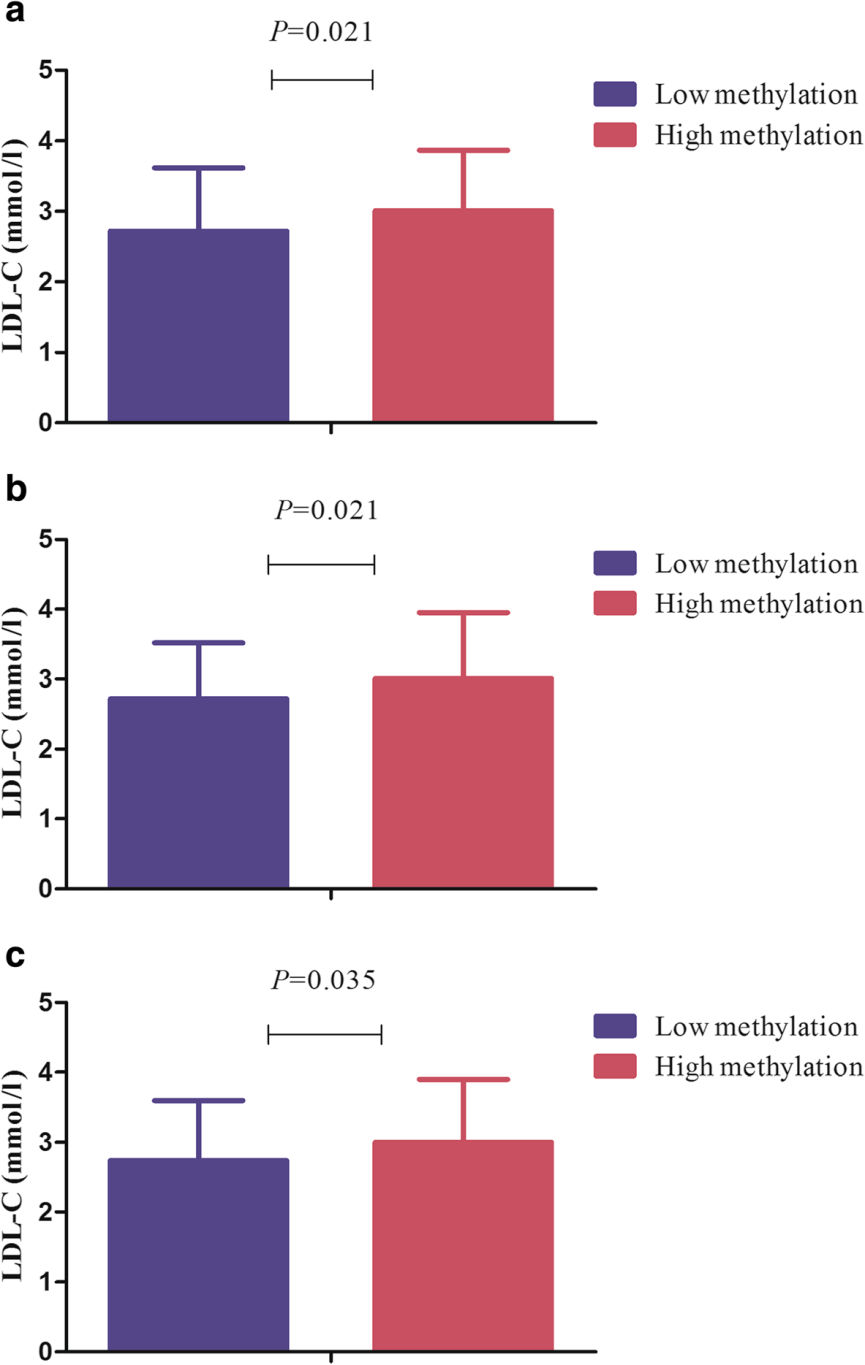
Association of methylation level of *C3* promoter with LDL-C levels. **a** CpG site 1; **b** CpG site 2; **c** average CpG sites

As shown in Fig. 3a-e**,** methylation levels of four CpG sites was positively associated with LDL-C levels (site 1: *r* = 0.152, *P* = 0.031; site 2: *r* = 0.191, *P* = 0.007; site 3: *r* = 0.176, *P* = 0.013; site 4: *r* = 0.166; *P* = 0.019). Additionally, the average methylation rate was also positively associated with LDL-C levels (*r* = 0.174, *P* = 0.014). After correcting for age, the correlation remained significant. According to the median of methylation levels, patients were divided into low methylation and high methylation groups. We observed the LDL-C levels in high methylation group were significantly higher than those in low methylation group for site 1, 2 and average (Fig. 4a-c). There was no correlation between methylation levels of CpG sites in *C3* gene and TC, TG and HDL-C levels (Additional file 4: Table S6).

## Discussion

To our knowledge, it was the first study to explore the relationship between methylation levels in *C3* promoter and CAD risk and lipid profiles.

DNA methylation is the most common and important epigenetic modification. General speaking, when the gene promoter CpG island is in hypermethylated status, its expression is inhibited; otherwise, the expression of the gene is up-regulated. However, there is also discrepancy in the association in some genes, such as ABCA1 [4, 15], PON1 [16, 17], etc. Unlike the changes in DNA itself, many epigenetic changes are reversible, which provides an optimistic outlook for the treatment of the diseases [18]. Environmental factors can affect the methylation of DNA promoter such as smoking [19], life stress [20], dietary [21], etc. For example, Park SL et al. found that internal smoking dose was associated with increased DNA methylation in circulating leukocytes at specific sites in Native Hawaiian smokers [19]. In a cross-sectional study conducted in 5186 Australian adult participants, the authors suggested that dietary intake of one-carbon metabolism nutrients was associated with blood DNA methylation [21].

In recent year, studies focusing on the relationship between epigenetics and CAD revealed that aberrant methylation of gene promoter might be implied in the etiology of CAD [6, 22]. As an important component of complement system, C3 may be involved in the occurrence and development of CAD by direct and indirect mechanisms. In 2018, Castellano-Castillo D et al. conducted a study to verify whether *C3* DNA methylation level in adipose tissues associated to body mass index (BMI) or obesity-associated metabolic diseases [10]. In their study, *C3* DNA methylation and C3 mRNA were detected in 60 participants who were divided into three groups by BMI value and mean methylation level for seven CpG sites in *C3* promoter was calculated. The results showed that *C3* methylation levels were positively associated with BMI and leptin. However, no significant association between *C3* DNA methylation and mRNA expression were observed.

In the present study, methylation levels of four CpG sites, located upstream of *C3* promoter, were measured. We failed to find an association between methylation of CpG sites in *C3* promoter and the risk of CAD. Subgroup analysis stratified by EH, DM and smoking status got the consistent results. Furthermore, seven haplotypes were established and no significant difference in haplotypes was observed between CAD and control groups. Interestingly, our study showed that *C3* DNA methylation levels were positively associated with LDL-C levels, although the Person correlation coefficient (*r* value) was relatively low. Unlike cancer samples, it is difficult to obtain coronary artery tissue. So, samples in most studies about DNA methylation and CAD were selected from blood cells [23]. The methylation status in different tissues from the same subjects was discrepancy [24]. The methylation level in blood cells might differ from that in vascular tissues. So, we should interpret the results cautiously.

There were several limitations in this study. First, the present study was a hospital-based case-control study. The diagnosis of control was based on the result of CAG and the criteria was luminal stenosis of main coronary artery less than 50%, which might lead to selection bias. Second, we did not detect the C3 levels because of the loss of serum samples. So, we did not determine whether the DNA methylation affected C3 expression. Third, only one CpG region was selected to determine the methylation of CpG sites. In addition, the sample size was relatively small in our study. Therefore, large sample and multi-center studies are needed to be performed to confirm our results. Finally, this was a retrospective study and the baseline data were consulted from the electronic medical record. Because of part of CAG images being not obtained, we could not analyze the relationship between CAD severity/degree of stenosis treated as the continuous variable and DNA methylation. Some environmental factors’ data, such as drinking dose, mental stress and economic condition, could not be obtained. Environmental factor might affect the DNA methylation, which has been well established. We could not analyze the influence of these factors on *C3* DNA methylation. Well designed and prospective study need to be investigated.

## Conclusions

In summary, the present study suggested that *C3* promoter methylation might not be associated with CAD risk. However, the methylation levels might be related to LDL-C levels.

## Additional files

Additional file 1: **Table S1.** Methylated CpG sites measured in this study. (DOCX 12.8 kb)

Additional file 2: **Table S2.** Primer sequences for C3 gene (start and end site were named as its relative distance to TSS). (DOCX 12.8 kb)

Additional file 3: **Table S3.** Subgroup analysis of methylation levels of CpG sites in C3 promoter between CAD and controls by smoking status. **Table S4.** Subgroup analysis of methylation levels of CpG sites in C3 promoter between CAD and controls by EH status. **Table S5.** Subgroup analysis of methylation levels of CpG sites in C3 promoter between CAD and controls by DM status. (ZIP 34.7 kb)

Additional file 4: **Table S6.** Relationship between methylation levels of CpG sites in C3 gene and TC, TG and HDL-C levels. (DOCX 13.1 kb)

## Abbreviations

C3: Component 3
CAD: Coronary artery disease
CAG: Coronary angiography
DM: Diabetes mellitus
EH: Essential hypertension
HDL-C: High-density lipoprotein cholesterol
LDL-C: Low-density lipoprotein cholesterol
TC: Total cholesterol
TG: Triglyceride

## References

[1] Shen CX, Ge JB (2018). Epidemic of cardiovascular disease in China. Current perspective and prospects for the future. Circulation.

[2] Lei HP, Yu XY, Wu H, Kang YH, Zhong WP, Cai LY, Zhang MZ, Chen JY, Mai LP, Ding QS, Yang M, Zhong SL (2018). Effects of PON1 Gene Promoter DNA Methylation and Genetic Variations on the Clinical Outcomes of Dual Antiplatelet Therapy for Patients Undergoing Percutaneous Coronary Intervention. Clin Pharmacokinet.

[3] Duan L, Liu C, Hu J, Liu Y, Wang J, Chen G, Li Z, Chen H (2018). Epigenetic mechanisms in coronary artery disease: The current state and prospects. Trends Cardiovasc Med.

[4] Ghaznavi H, Mahmoodi K, Soltanpour MS (2018). A preliminary study of the association between the *ABCA1* gene promoter DNA methylation and coronary artery disease risk. Mol Biol Res Commun.

[5] Nakatochi M, Ichihara S, Yamamoto K, Naruse K, Yokota S, Asano H, Matsubara T, Yokota M (2017). Epigenome-wide association of myocardial infarction with DNA methylation sites at loci related to cardiovascular disease. Clin Epigenetics.

[6] Li J, Zhu X, Yu K, Jiang H, Zhang Y, Deng S, Cheng L, Liu X, Zhong J, Zhang X, He M, Chen W, Yuan J, Gao M, Bai Y, Han X, Liu B, Luo X, Mei W, He X, Sun S, Zhang L, Zeng H, Sun H, Liu C, Guo Y, Zhang B, Zhang Z, Huang J, Pan A, Yuan Y, Angileri F, Ming B, Zheng F, Zeng Q, Mao X, Peng Y, Mao Y, He P, Wang QK, Qi L, Hu FB, Liang L, Wu T (2017). Genome-Wide Analysis of DNA Methylation and Acute Coronary Syndrome. Circ Res.

[7] Ursini F, Abenavoli L (2018). The emerging role of complement C3 as a biomarker of insulin resistance and cardiometabolic diseases: preclinical and clinical evidence. Rev Recent Clin Trials.

[8] Jiang H, Guo M, Dong L, Cao C, Wang D, Liang X, Guo F, Xing Z, Bu P, Liu J (2014). Levels of acylation stimulating protein and the complement component 3 precursor are associated with the occurrence and development of coronary heart disease. Exp Ther Med.

[9] King R, Tiede C, Simmons K, Fishwick C, Tomlinson D, Ajjan R (2015). Inhibition of complement C3 and fibrinogen interaction: a potential novel therapeutic target to reduce cardiovascular disease in diabetes. Lancet.

[10] Castellano-Castillo D, Moreno-Indias I, Fernandez-Garcia JC, Clemente-Postigo M, Castro-Cabezas M, Tinahones FJ, Queipo-Qrtuño MI, Cardona F (2018). Complement factor C3 methylation and mRNA expression is associated to BMI and insulin resistance in obesity. Genes (Basel).

[11] Cai G, Zhang B, Shi G, Weng W, Yang L, Xue S (2016). Endothelial lipase genetic polymorphisms and lipid-lowering response in patients with coronary artery disease on rosuvastatin. Lipids Health Dis.

[12] Cai G, Zhang B, Ma C, Shi G, Weng W, Xue S (2016). Associations of rs3744841 and rs3744843 polymorphisms in endothelial lipase gene with risk of coronary artery disease and lipid levels in a Chinese population. PLoS One.

[13] Zhou S, Cai B, Zhang Z, Zhang Y, Wang L, Liu K, Zhang H, Sun L, Cai H, Lu G, Liu X, Xu G (2017). CDKN2B methylation and aortic arch calcification in patients with ischemic stroke. J Atheroscler Thromb.

[14] Pu W, Wang C, Chen S, Zhao D, Zhou Y, Ma Y, Wang Y, Li C, Huang Z, Jin L, Guo S, Wang J, Wang M (2017). Targeted bisulfite sequencing identified a panel of DNA methylation-based biomarkers for esophageal squamous cell carcinoma (ESCC). Clin Epigenetics.

[15] Talens RP, Jukema JW, Trompet S, Kremer D, Westendorp RG, Lumey LH, Sattar N, Putter H, Slagboom PE, Heijmans BT, PROSPER Group (2012). Hypermethylation at loci sensitive to the prenatal environment is associated with increased incidence of myocardial infarction. Int J Epidemiol.

[16] Gómez-Uriz AM, Goyenechea E, Campión J, de Arce A, Martinez MT, Puchau B, Milagro FI, Abete I, Martínez JA, Lopez de Munain A (2014). Epigenetic patterns of two gene promoters (TNF-α and PON) in stroke considering obesitycondition and dietary intake. J Physiol Biochem.

[17] Fiorito G, Guarrera S, Valle C, Ricceri F, Russo A, Grioni S, Mattiello A, Di Gaetano C, Rosa F, Modica F, Iacoviello L, Frasca G, Tumino R, Krogh V, Panico S, Vineis P, Sacerdote C, Matullo G (2014). B-vitamins intake, DNA-methylation of one carbon metabolism and homocysteine pathway genes and myocardial infarction risk: the EPICOR study. Nutr Metab Cardiovasc Dis.

[18] Xu S, Pelisek J, Jin ZG. Atherosclerosis is an epigenetic disease. Trends Endocrinol Metab. 2018. 10.1016/j.tem.2018.04.007 PMID: 29753613. 10.1016/j.tem.2018.04.007 PMC7202861 29753613

[19] Park SL, Patel YM, Loo LWM, Mullen DJ, Offringa IA, Maunakea A, Stram DO, Siegmund K, Murphy SE, Tiirikainen M, Le Marchand L (2018). Association of internal smoking dose with blood DNA methylation in three racial/ethnic populations. Clin Epigenetics.

[20] Santos HP, Nephew BC, Bhattacharya A, Tan X, Smith L, Alyamani RAS, Martin EM, Perreira K, Fry RC, Murgatroyd C (2018). Discrimination exposure and DNA methylation of stress-related genes in Latina mothers. Psychoneuroendocrinology.

[21] Chamberlain JA, Dugué PA, Bassett JK, Hodge AM, Brinkman MT, Joo JE, Jung CH, Makalic E, Schmidt D F, Hopper JL, Buchanan DD, English DR, Southey MC, Giles GG, Milne RL. Dietary intake of one-carbon metabolism nutrients and DNA methylation in peripheral blood. Am J Clin Nutr. 2018. 10.1093/ajcn/nqy119 PMID:30101351. 10.1093/ajcn/nqy119 30101351

[22] Li D, Yan J, Yuan Y, Wang C, Wu J, Chen Q, Song J, Wang J (2018). Genome-wide DNA methylome alterations in acute coronary syndrome. Int J Mol Med.

[23] Fernández-Sanlés A, Sayols-Baixeras S, Subirana I, Degano IR, Elosua R. Association between DNA methylation and coronary heart disease or other atherosclerotic events: a systematic review. Atherosclerosis. 2017;263:325–33. 10.1016/j.atherosclerosis.2017.05.022 PMID: 28577936. 10.1016/j.atherosclerosis.2017.05.022 28577936

[24] Thomas M, Knoblich N, Wallisch A, Glowacz K, Becker-Sadzio J, Gundel F, Brückmann C, Nieratschker V (2018). Increased BDNF methylation in saliva, but not blood, of patients with borderline personality disorder. Clin Epigenetics.

