# Supplementary material for: A preliminary study showing no association between methylation levels of C3 gene promoter and the risk of CAD
Source: Lipids Health Dis. 2019 Jan 5;18:5. doi: 10.1186/s12944-018-0949-4 (PMC6320636; doi:10.1186/s12944-018-0949-4)
Supplement: Supplementary file 1 — Table S1. Methylated CpG sites measured in this study. (DOCX 12.8 kb) [file 12944_2018_949_MOESM1_ESM.docx]

**Supplemental table 1. Methylated CpG sites measured in this study**

| **Position** | **Genomic location＊** | **Relative to TSS, bp** |
| --- | --- | --- |
| 1 | 6721741 | -1048 |
| 2 | 6721749 | -1056 |
| 3 | 6721757 | -1064 |
| 4 | 6721769 | -1076 |

＊The chromosomal location of each CpG site according to assembly GRCh37/hg19
