# Supplementary material for: A preliminary study showing no association between methylation levels of C3 gene promoter and the risk of CAD
Source: Lipids Health Dis. 2019 Jan 5;18:5. doi: 10.1186/s12944-018-0949-4 (PMC6320636; doi:10.1186/s12944-018-0949-4)
Supplement: Supplementary file 2 — Table S2. Primer sequences for C3 gene (start and end site were named as its relative distance to TSS). (DOCX 12.8 kb) [file 12944_2018_949_MOESM2_ESM.docx]

**Supplemental table 2. Primer sequences for C3 gene (start and end site were named as its relative distance to TSS)**

| Gene | Chr | PCR size (bp) | Start site | End site | Primer |
| --- | --- | --- | --- | --- | --- |
| C3 | 19 | 161 | -1109 | -948 | F: TTTGTTATTTAGGTTAAATTGTAGTGGTGT  R: ACCCCCATCTCCACAAAAA |

C3, component 3; Chr,chromosome; PCR,polymerase chain reaction.
