# Supplementary material for: A preliminary study showing no association between methylation levels of C3 gene promoter and the risk of CAD
Source: Lipids Health Dis. 2019 Jan 5;18:5. doi: 10.1186/s12944-018-0949-4 (PMC6320636; doi:10.1186/s12944-018-0949-4)
Supplement: Supplementary file 3 — Table S3. Subgroup analysis of methylation levels of CpG sites in C3 promoter between CAD and controls by smoking status. Table S4. Subgroup analysis of methylation levels of CpG sites in C3 promoter between CAD and controls by EH status. Table S5. Subgroup analysis of methylation levels of CpG sites in C3 promoter between CAD and controls by DM status. (ZIP 34.7 kb) [file 12944_2018_949_MOESM3_ESM.zip › Supplemental table 3.docx]

**Supplemental table 3. Subgroup analysis of methylation levels of CpG sites in *C3* promoter between CAD and controls by smoking status**

| **CpG site** | **Smoking** | | | | |  | **Non-smoking** | | | | |
| --- | --- | --- | --- | --- | --- | --- | --- | --- | --- | --- | --- |
|  | **CAD (%)** |  | **Control (%)** | ***P*** | ***P^a^*** |  | **CAD (%)** |  | **Control (%)** | ***P*** | ***P^a^*** |
| 1 | 54.22±8.79 |  | 55.00±7.09 | 0.590 | 0.394 |  | 54.98±7.23 |  | 57.52±5.90 | 0.099 | 0.053 |
| 2 | 55.92±8.44 |  | 56.15±7.33 | 0.874 | 0.678 |  | 55.81±7.15 |  | 58.66±5.87 | 0.065 | 0.031 |
| 3 | 59.78±7.74 |  | 59.98±6.88 | 0.879 | 0.704 |  | 59.85±7.05 |  | 62.63±6.02 | 0.073 | 0.035 |
| 4 | 58.33±8.28 |  | 58.53±7.29 | 0.891 | 0.687 |  | 58.10±6.70 |  | 60.99±5.85 | 0.054 | 0.022 |
| Average | 57.06±8.19 |  | 57.41±7.07 | 0.800 | 0.600 |  | 57.18±7.00 |  | 59.95±5.82 | 0.069 | 0.032 |

C3, component 3; CAD, coronary artery disease; *P*^a^, adjustment for EH, DM, and smoking status.
