# Supplementary material for: A preliminary study showing no association between methylation levels of C3 gene promoter and the risk of CAD
Source: Lipids Health Dis. 2019 Jan 5;18:5. doi: 10.1186/s12944-018-0949-4 (PMC6320636; doi:10.1186/s12944-018-0949-4)
Supplement: Supplementary file 4 — Table S6. Relationship between methylation levels of CpG sites in C3 gene and TC, TG and HDL-C levels. (DOCX 13.1 kb) [file 12944_2018_949_MOESM4_ESM.docx]

**Supplemental table 6. Relationship between methylation levels of CpG sites in C3 gene and TC, TG and HDL-C levels**

| **CpG site** |  | **TC** | **TG** | **HDL-C** |
| --- | --- | --- | --- | --- |
| 1 | r | 0.091 | -0.027 | -0.055 |
|  | *P* | 0.201 | 0.702 | 0.441 |
| 2 | r | 0.116 | -0.029 | -0.066 |
|  | *P* | 0.102 | 0.685 | 0.354 |
| 3 | r | 0.097 | -0.031 | -0.083 |
|  | *P* | 0.173 | 0.667 | 0.241 |
| 4 | r | 0.085 | -0.046 | -0.076 |
|  | *P* | 0.233 | 0.514 | 0.282 |
| Average | r | 0.098 | -0.034 | -0.071 |
|  | *P* | 0.166 | 0.636 | 0.319 |

C3, component 3; TC, total cholesterol; TG, triglyceride; HDL-C, high-density lipoprotein cholesterol.
